# SARS-CoV-2: Immunopeptidomics and Other Immunological Studies

**DOI:** 10.3390/vaccines10111975

**Published:** 2022-11-21

**Authors:** Vivek P. Chavda, Elrashdy M. Redwan

**Affiliations:** 1 Department of Pharmaceutics and Pharmaceutical Technology, L M College of Pharmacy, Ahmedabad 380008, India; 2 Biological Science Department, Faculty of Science, King Abdulaziz University, P.O. Box 80203, Jeddah 21589, Saudi Arabia; 3 Protein Research Department, Genetic Engineering and Biotechnology Research Institute, City of Scientific Research and Technological Applications (SRTA City), Alexandria 21934, Egypt

Severe acute respiratory syndrome coronavirus 2 (SARS-CoV-2) has produced a significant continuing epidemic worldwide [1]. The comprehensive identification of antigen sequences for T-cell immuno-surveillance considerably improves our understanding and modulation of humoral responses to viral infection or active vaccination. It facilitates long-term immunization against viruses and targets tissue to govern the safety and efficacy of vaccines by increasing the durability of CD4+ and CD8+ cell response [2,3,4,5,6]. On antigen-presenting cells (APCs) from a panel of healthy donors chosen to reflect the majority of gene use from this highly polymorphic molecule, mass spectrometry is utilized to discover 526 distinct sequences of the extracellular domain of the SARS-CoV-2 spike glycoprotein in combination with human leukocyte antigen class II molecules [7]. Immunopeptidomics is the study of employing mass spectrometry to investigate the structure and kinetics of peptides expressed by major histocompatibility complex (MHC) class I and class II molecules (MS). MHC class I and class II provide peptides for identification by CD8+ and CD4+ T cells, respectively, and are loaded with peptides via different antigen processing procedures previously studied in depth. MHC molecules are genetically variable, with various alleles preferring distinct binding sets of peptide sequences [8]. Three distinct HLA loc1 construct the alpha chain of MHC class I for at least six alleles in each individual. Furthermore, three loc1 encode an MHC class II alpha and a beta chain. To completely detect pathogen-derived antigens shown on MHCs, whole-proteome-scale techniques are required due to the richness and unpredictability of the immune peptidome [9]. MS studies of MHC peptides may be performed in two ways. Without prior knowledge of the sample’s constitution, peptides are recognized and selected for mass spectrum capture in a data-dependent mode, allowing the discovery of new vaccine targets [10]. Researchers may examine the mechanics and process of antigen presentation using the targeted model since it provides consistent detection and quantification across several samples. A specified list of targets is chosen for collection [11]. Scientists from various scientific fields have shown interest in MS-based immune-peptidomics due to their significance in identifying T-cell targets against cancer and, more recently, pandemic diseases [12,13]. Even though the immune-peptidomics field has historically been restricted to a few research organizations, its consistent growth may signal its direction toward large-scale implementation soon. About 25 researchers (totaling 1197 participants) have looked at T-cell-mediated immune responses to SARS-CoV-2 to identify potential T-cell epitopes generated from the SARS-CoV-2 proteome [14,15]. Immunopeptidome analysis of high-containment viruses is technically hard and necessitates using techniques that accomplish total viral inactivation while preserving the HLA-peptide complex’s integrity [7]. Understanding the immune system and guiding the creation of next-generation vaccines and immunotherapies against autoimmunity, infectious illnesses, and malignancies requires comprehending the structure of the human immunopeptidome [7,16]. The only technique capable of accurately, methodically, and objectively examining the immune peptidome up to this point is MS. As a result, developing sophisticated analytical MS procedures is critical for understanding the immune peptidome with increasing depth and robustness [7]. The challenge of mapping the viral immune-peptidome might be complicated by unclear spectral assignments, particularly with the host antigen-derived peptide background, which typically demands thorough validation of peptide spectra using synthetic peptides. An accessible and thorough online library of SARS-CoV-2 peptides contains 39,650 synthetic peptides produced by a thorough and varied proteolytic digestion of peptide precursors drawn from the viral proteome [2,17], which facilitates quick SARS-CoV-2 peptide validation. Full annotations of these synthetic peptides are free on VirusMS, including basic experimental details, peptide HLAI binding prediction, third-party database cross-referencing, and complete spectrum data [18,19]. With the SARS-CoV-2 spike glycoprotein, this study provides an accurate and thorough immune-peptidomics examination that enables an in-depth assessment of characteristics that may help with vaccine development to stop the ongoing COVID-19 pandemic [17].

Several strategies are acceptable alternatives in an epidemic crisis, including medication repurposing, immunization, and immunotherapy. Peptide targets are used in immunotherapy and vaccination [20,21,22]. Due to their quicker and less expensive production, peptides and smaller pieces of proteins are preferred. The benefits of using peptides as medications include their quick discovery, affinity, specificity for specific targets, and minimal toxicity due to the remote chance of accumulating in the body [23]. Peptides were first regarded as poor therapeutic choices because of their costly and inefficient production processes, low bioavailability, and limited stability against proteolysis by peptidases in the blood and gastrointestinal tract [24].

Immunopeptidomics screenings based on mass spectrometry enable the detection of viral/bacterial antigens shown on infected cells (Figure 1) [2,25]. These antigens may readily encode in next-generation nucleic acid-based vaccinations as innovative antiviral/microbial-resistance-combating weapons. Recent advances in mass-spectrometry-based proteomics have resulted in the development of immune-peptidomics approaches, which allow for the untargeted identification of viral epitopes exhibited on the surface of infected cells. Immunopeptidomics offers enormous promise for identifying antigens that might be encoded in vaccines based on viral vectors or nucleic acids [26,27,28,29]. An analysis of the proteome of infected cells demonstrated that early expressed viral proteins had a greater impact on HLA-I presentation and immunogenicity [7]. Immunopeptidomic research has contributed to the comprehension of antigen presentation and T-cell priming in the setting of infection [30]. A panel of healthy donors was analyzed using mass spectrometry to identify over 500 distinct sequences of SARS-CoV-2 spike glycoprotein extracellular domains combined with human leukocyte antigen class II molecules on antigen-presenting cells [31]. The investigators recovered T-cell replicas that responded widely to the spike protein of other coronaviruses, suggesting that preexisting cross-reactive memory T-cells are recalled after infection with SARS-CoV-2 [32].

The effectiveness of proteomics technologies helps elucidate disease mechanisms and provide COVID-19 prognostic indicators, as well as suggests that proteomics methods may be crucial in understanding novel variants and their changed disease pathophysiology [23,33].

A crucial method for identifying variant peptides is mass spectrometry. For example, high-density viral proteome microarrays can be utilized to detect specific immunological signatures to develop serological diagnostic tools against novel variants [34]. Immunopeptidomics has aided in the development of broad-spectrum SARS-CoV-2 T-cell vaccine candidates and has provided deeper insights into the T-cell response to viral infection when combined with mass-spectrometry-based methods [35].

## Figures and Tables

**Figure 1:**
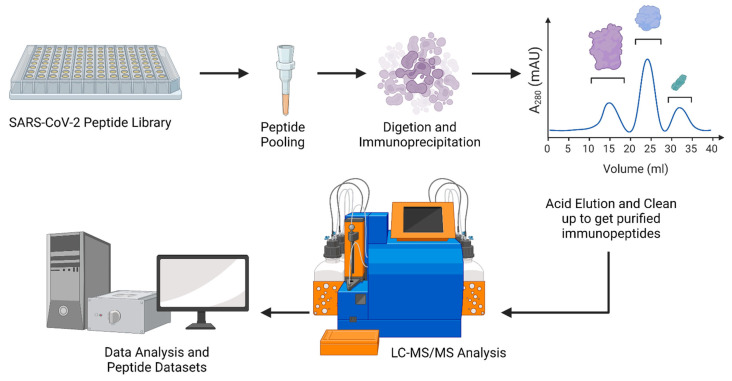
Overview of the immunepeptidomics screenings for COVID-19 (created with Biorender.com (access on 18 November 2022)).
